# Alpelisib Plus Fulvestrant or Letrozole Demonstrates Sustained Benefits Across Subgroups of Patients with *PIK3CA*-Mutated HR+/HER2– Advanced Breast Cancer

**DOI:** 10.1093/oncolo/oyac011

**Published:** 2022-03-28

**Authors:** Anne Jacobson

## Abstract

Alpelisib and endocrine therapy provides ongoing treatment benefits for patients with HR-positive, HER2-negative, *PIK3CA*-mutated advanced breast cancer, according to updated findings from all 3 cohorts of the phase II BYLieve trial.

Standard first-line treatment for hormone receptor (HR)-positive, human epidermal growth factor receptor 2 (HER2)-negative advanced breast cancer is endocrine therapy in combination with a cyclin dependent kinase (CDK) 4/6 inhibitor. Resistance to endocrine therapy and disease progression are common, leading to the need for subsequent-line therapy. Moreover, mutations in the PIK3 catalytic subunit alpha (*PIK3CA*) gene, which occur in approximately 40% of patients with HR-positive, HER2-negative breast cancer, predict worse survival outcomes.

The phase II BYLieve trial is the first prospective study to evaluate alpelisib plus endocrine therapy (fulvestrant or letrozole) in patients with HR-positive, HER2-negative, *PIK3CA*-mutated advanced breast cancer who have progressed on or after prior treatment with a CDK4/6 inhibitor.^1–5^ Patients enrolled in BYLieve (*N* = 336) were assigned to study cohorts (A-C) based on their most immediate prior treatment history (Table 1).

**Table 1.** BYLieve patient cohorts and treatment assignments

Cohort	Immediate prior therapy	Study treatment
A (*n* = 112)	CDK4/6 inhibitor and an aromatase inhibitor	Alpelisib 300mg once daily plus fulvestrant 500 mg
B (*n* = 112)	CDK4/6 inhibitor and fulvestrant	Alpelisib 300mg once daily plus letrozole 2.5 mg
C (*n* = 112)	Chemotherapy or endocrine therapy	Alpelisib 300mg once daily plus fulvestrant 500 mg

Multiple updates from the BYLieve trial provide insights on the optimal management of these patients, including the potential role of clinical and genetic biomarkers in selecting treatment plans and predicting response to therapy.^1–5^

## Cohort A Update: Alpelisib Plus Fulvestrant After Prior Therapy with a CDK4/6 Inhibitor and an AI

Results from an updated BYLieve Cohort A analysis demonstrate the ongoing benefit of alpelisib plus fulvestrant in patients with HR-positive, HER2-negative, *PIK3CA*-mutated advanced breast cancer whose disease progressed on or after treatment with a CDK4/6 inhibitor plus an aromatase inhibitor.^1^

With 18 months of follow-up, 22.2% of patients remained free from disease progression or death. The median overall survival was 26.4 months and the median progression-free survival (PFS) was 7.3 months.

The overall response rate (ORR) was 19.0%, with 0.8% of patients achieving a complete response and 18.2% achieving a partial response. The median duration of response was 13.8 months. In the analysis of tumor reduction, 70.1% of patients had a reduction in tumor size from baseline that was maintained through the 6-month follow-up assessment.

## Cohort C Update: Alpelisib Plus Fulvestrant After Prior Chemotherapy or Endocrine Therapy

An updated analysis from BYLieve Cohort C examined outcomes among patients whose cancer progressed on or after treatment with an aromatase inhibitor and who received chemotherapy or endocrine therapy as immediate prior treatment.^2^ Two-thirds of patients (67.5%) received prior treatment with a CDK4/6 inhibitor as well.

At the 6-month assessment, 48.7% of patients treated with alpelisib plus fulvestrant were alive without disease progression. The median PFS was 5.6 months. At 6 months, 65.1% of patients experienced a decrease in tumor size from baseline. The ORR was 24.3%.

Findings from the BYLieve Cohort C updated analysis support the clinical benefit of alpelisib plus fulvestrant in patients with HR-positive, HER2-negative, *PIK3CA*-mutated advanced breast cancer who were treated primarily in the third-line setting.

## Clinical Biomarker: Duration of Prior CDK4/6 Inhibitor Therapy

Another subgroup analysis examined PFS following treatment with alpelisib plus fulvestrant/letrozole by the duration of prior CDK4/6 inhibitor therapy.^3^ The median PFS following treatment with alpelisib plus fulvestrant (Cohort A) was 12.0 months for patients who discontinued prior CDK4/6 inhibitor therapy within 6 months, compared with 6.2 months among those who remained on prior therapy for longer than 6 months (HR, 0.51; 95% CI, 0.29-0.89) (Table 2).

**Table 2.** Median progression-free survival by duration of prior CDK4/6 inhibitor therapy

Endpoints	≤6 months	>6 months	HR (95% CI)
Cohort A	(*n* = 26)	(*n* = 94)	0.51 (0.29-0.89)
PFS events	57.7%	79.8%	
Median PFS	12.0 months	6.2 months	
Cohort B	(*n* = 30)	(*n* = 83)	0.72 (0.45-1.18)
PFS events	76.7%	77.1%	
Median PFS	5.9 months	5.6 months	

In contrast, there was no interaction between PFS following alpelisib plus letrozole and duration of prior CDK4/6 inhibitor therapy (Cohort B). Together, these findings support the use of alpelisib plus endocrine therapy as an immediate next-line option for patients whose disease progresses on or after prior CDK4/6 inhibitor therapy.

## Genetic Biomarker: ctDNA Fraction

Another exploratory biomarker analysis examined potential predictors of treatment benefit among baseline biomarkers in patients in Cohorts A and B of the BYLieve trial.^4^ The analysis showed that alpelisib in combination with fulvestrant or letrozole is effective regardless of tumor genomic profile. However, there is an association between improved PFS and

- Low circulating tumor DNA (ctDNA) fraction, defined as <10% or undetectable
- Low tumor mutation burden (TMB), defined as <10 mutations/Mb
- Absence of amplifications in chromosome 8 and/or 11.

In Cohort C, lower ctDNA fraction significantly predicted better PFS outcomes.^2^ The median PFS was 16.7 months in patients with low ctDNA fraction, compared with 5.4 months in patients with higher ctDNA (HR, 0.31; *p* =.00052).

## Genetic Biomarker: *ESR1* Mutation

Researchers also evaluated the relationship between PFS in patients treated with alpelisib plus fulvestrant/letrozole and *ESR1* mutations detected via ctDNA, finding no interaction in Cohorts A or C.^2,5^ In contrast, in Cohort B, there was a numerical trend toward *ESR1* mutations and shorter PFS.^5^ These findings suggest that treatment with alpelisib plus fulvestrant may be the preferred option for patients with HR-positive, HER2-negative, *PIK3CA*-mutated advanced breast cancer when the presence of an *ESR1* mutation is suspected.

In summary, extended follow-up from the phase II BYLieve study support the sustained benefit of alpelisib plus endocrine therapy in patients with HR-positive, HER2-negative, *PIK3CA*-mutated advanced breast cancer. Future BYLieve updates will provide further insights on *PIK3CA*-targeted treatment in this patient population, including the roles of clinical and genetic biomarkers in individualized therapy.

## Notes

Please note that this content was created for the purpose of summarizing the content of the San Antonio Breast Cancer Symposium 2021 and has not been peer reviewed under the standard policies of *The Oncologist*.
